# Temporal Dynamics of Stress-Induced Alternations of Intrinsic Amygdala Connectivity and Neuroendocrine Levels

**DOI:** 10.1371/journal.pone.0124141

**Published:** 2015-05-06

**Authors:** C. W. E. M. Quaedflieg, V. van de Ven, T. Meyer, N. Siep, H. Merckelbach, T. Smeets

**Affiliations:** Faculty of Psychology and Neuroscience, Maastricht University, Maastricht, The Netherlands; National University of Singapore, SINGAPORE

## Abstract

Stress-induced changes in functional brain connectivity have been linked to the etiology of stress-related disorders. Resting state functional connectivity (rsFC) is especially informative in characterizing the temporal trajectory of glucocorticoids during stress adaptation. Using the imaging Maastricht Acute Stress Test (iMAST), we induced acute stress in 39 healthy volunteers and monitored the neuroendocrine stress levels during three runs of resting state functional magnetic resonance imaging (rs-fMRI): before (run 1), immediately following (run 2), and 30min after acute stress (run 3). The iMAST resulted in strong increases in cortisol levels. Whole-brain analysis revealed that acute stress (run 2 - 1) was characterized by changes in connectivity of the amygdala with the ventrolateral prefrontal cortex (vlPFC), ventral posterior cingulate cortex (PCC), cuneus, parahippocampal gyrus, and culmen. Additionally, cortisol responders were characterized by enhanced amygdala - medial prefrontal cortex (mPFC) connectivity. Stress recovery (run 3 - 2) was characterized by altered amygdala connectivity with the dorsolateral prefrontal cortex (dlPFC), ventral and dorsal anterior cingulate cortex (ACC), anterior hippocampal complex, cuneus, and presupplementary motor area (preSMA). Opposite to non-responders, cortisol responders were characterized by enhanced amygdala connectivity with the anterior hippocampal complex and parahippocampal gyrus, and reduced connectivity with left dlPFC, dACC, and culmen during early recovery. Acute stress responding and recovery are thus associated with changes in the functional connectivity of the amygdala network. Our findings show that these changes may be regulated via stress-induced neuroendocrine levels. Defining stress-induced neuronal network changes is pertinent to developing treatments that target abnormal neuronal activity.

## Introduction

Dysfunction of neuroendocrine regulation and impaired coping abilities have been implicated in a variety of psychiatric disorders (e.g., depression, anxiety). Acute stress regulated by the neuroendocrine system affects brain activity and, hence, influences the capacity to cope with stress. Glucocorticoids bind to mineralocorticoid and glucocorticoid receptors (MR and GR, respectively) in the brain and exert a time [1] and spatial [2,3,4,5,6] specific mode of action, enabling the prioritisation of adaptive cognitive processes after having experienced a stressor. In the immediate phase, stress elicits an emotional response expressed as subjective withdrawal motivation as well as enhanced vigilance, perception and attentional focusing on threat-related stimuli. This phase is focused on promoting survival. Subsequently, processes are initiated directed at restoring homeostasis, such as emotion regulation [7,8]. To date, only few studies have investigated the activation of distinct brain networks during acute stress and recovery [9,10] and its relationship to neuroendocrine stress markers [11,12]. This is partly due to fact that it is exceedingly challenging to effectively elicit neuroendocrine stress responses in the constraints of a neuroimaging environment [13]. The paradigms used until now resulted in relatively modest cortisol increases, making it difficult to address how glucocorticoids change brain activation patterns after a stressor.

The functional connectivity of brain areas has been investigated using task absent (i.e., resting state) functional magnetic resonance imaging measurements (rs-fMRI); [14]. Resting state functional connectivity (rsFC) is especially informative when studying the effects of post-stress brain activation changes. rsFC parameters are not related to a task, making it possible to explore the diffuse effects of stress on the brain. Moreover, connectivity alterations following stress and its relationship to neuroendocrine stress markers are particularly intriguing in light of the suggested role of glucocorticoids in stress adaptation [15] and the etiology of stress-related disorders [9,10].

The amygdala is one of the first brain areas to react to a stressor. It initiates the autonomic nervous system (ANS) and hypothalamic-pituitary-adrenal (HPA) responses, thereby mediating the initial surge in vigilance and optimizing the detection of threats to homeostasis [7,16,17]. Moreover, the amygdala is crucially involved in stress induced long-term adaptive responses such as enhanced memory consolidation [18,19,20]. The medial prefrontal cortex (mPFC) is involved in mediating amygdala activity during regulation of autonomic and affective responses [21–25]. Previous neuroimaging studies demonstrated time specific enhancement of the functional connectivity of the amygdala with PFC areas during the acute stress [9] and recovery [10] phase.

The current study investigated the moderating role of glucocorticoids on the *change* in amygdala rsFC during two phases that follow stress exposure: the acute and early recovery phases. Stress was induced using the imaging Maastricht Acute Stress Test (iMAST) [26], a neuroimaging stress task that has been shown to generate considerable subjective stress as well as robust increases in glucocorticoid stress hormones (e.g., the primary human glucocorticoid cortisol). Based on previous studies investigating inter-individual differences in stress reactivity [9,27,28], we also compared cortisol responders and non-responders in their change of amygdala rsFC and its relationship to neuroendocrine stress markers. It is hypothesized that the acute stress phase is characterized by connectivity changes with areas involved in vigilance and perception, while early recovery is characterized by connectivity changes with areas involved in emotion regulation. Furthermore, amygdala connectivity with the mPFC is hypothesized to differentiate between cortisol responders and non-responders.

## Materials and Methods

### Participants

The sample of the current study consisted of 42 right-handed, scanner-naïve participants (21 men, 21 women, mean age = 21.8 years, *SD* = 2.1; range: 18–35 years) see also [26]. All participants underwent a screening protocol assessing their physical and mental health, fMRI aptness, and handedness (see experimental procedures in S1 File). Test protocols were approved by the standing ethics committee of the Faculty of Psychology and Neuroscience, Maastricht University. Participants signed a written informed consent form and were given a small monetary reward.

### Study procedure

**Fig 1:**
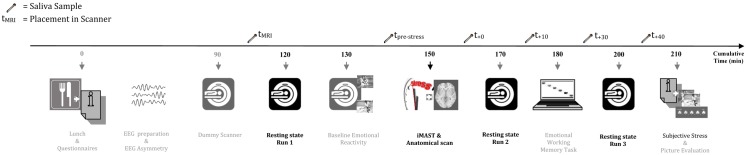
Overview of the study procedure. Abbreviation: iMAST: imaging Maastricht Acute Stress Test.

An overview of the experiment is shown in Fig 1. The three resting state measures were part of a larger study investigating the neural correlates of resilience (see S1 file) and in [26].

One and a half hour after arrival, participants received general information about fMRI measures and were prepared for a 30min simulation scan. To reduce anticipatory anxiety for, and familiarization with the scanner environment, participants were extensively trained in a dummy scanner. Subsequently, participants were placed in the scanner and a baseline cortisol sample (t_MRI_) was taken. The MRI session lasted for about 1.5h and consisted of the following runs (in chronological order): resting state before stress induction (duration: 8min), implicit emotion task (16min), iMAST including anatomical scan (15min), resting state immediate after stress induction (8min), emotional working memory task (18min), and resting state 30min after stress induction (8min). During the resting state scan, a fixation point was presented. Participants were instructed to relax, not think of anything in particular and keep their eyes open. In total, six saliva samples were collected during scanning (cf. supra).

The implicit emotion task was used as empirical localizer for left and right amygdala seeds. Participants completed two blocks of the task in which they were asked to decide whether 84 pictures (International Affective Picture System; IAPS) [29]—subdivided into the categories neutral, positive or negative—were situated in- or outdoors via a button press (see S1 file).

### Stress induction equipment and procedures

The imaging Maastricht Acute Stress Test (iMAST) [26] consists of a 5min preparation phase in which the task is explained and a 10min acute stress phase that includes several exposures to cold pressor stress (i.e., stimuli of 2°C) and mental arithmetic challenges (i.e., counting backwards as fast and accurate as possible in steps of 17 starting at 2043) along with social-evaluative pressure (i.e., negative feedback). The iMAST protocol increases unpredictability and uncontrollability by applying without prior warning one, two or three hot pulses (49°C) of 10s during each of the mental arithmetic trials, and by telling participants that the computer would randomly choose the order and duration of the cold pressor and mental arithmetic trials (see S1 file).

### Subjective, neuroendocrine and physiological stress responses

#### Subjective stress

A 100 mm visual analogue scale (VAS) was used at the end of the imaging session to assess subjective stress related to the iMAST. Participants had to specify their level of agreement with the statement on how stressful they had felt during the iMAST (anchors: 0 =“*not at all*”; 100 = “*extremely*”).

#### Cortisol

Neuroendocrine stress prior to and in response to the iMAST was measured via saliva samples that were obtained with synthetic Salivettes (Sarstedt, Etten-Leur, The Netherlands). Participants provided saliva samples immediately after having been placed in the MRI scanner (t_MRI_), 5min before the iMAST (t_pre-stress_ i.e., 25min after the t_MRI_ sample was taken) and 4 times after stress exposure (t_+0_, t_+10_, t_+30_, t_+40_ with reference to the end of the stressor). For each participant individually, the Area Under the Curve with respect to increase (AUCi) was calculated as a single measure of the total cortisol concentration in response to the iMAST [30]. Two male participants did not provide enough saliva via the Salivettes to be analyzed. One clear cortisol outlier (> 3*SD* above the mean) was excluded from all analyses. Thus, the final sample consisted of 39 participants.

**Fig 2:**
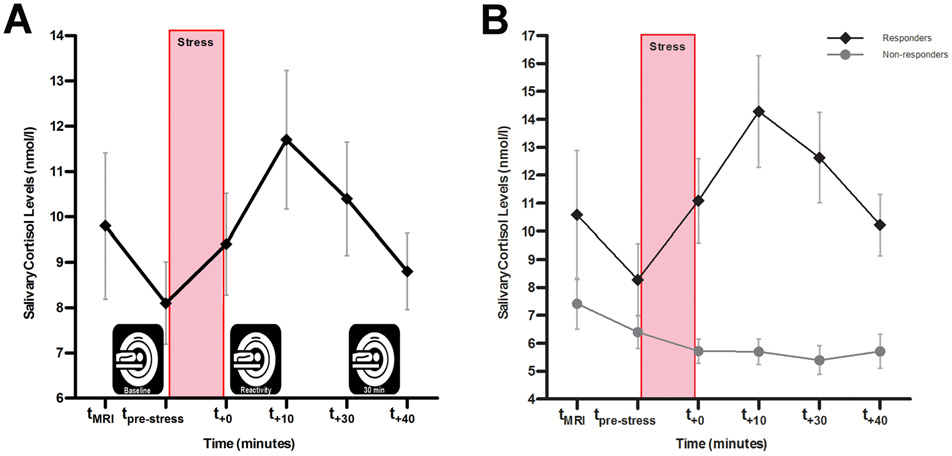
Neuroendocrine responses to the imaging Maastricht Acute Stress Test (iMAST). **A)** Cortisol response with respect to the imaging runs. **B)** Cortisol responses separately for responders and non-responders. Values represent (untransformed) means ± *SEM*.

Based on previous work [31], delta cortisol increases (i.e., peak cortisol level after the iMAST minus pre-stress cortisol level) of 1.5 nmol/l or larger were used to distinguish between cortisol responders (*n* = 27) and non-responders (*n* = 12, see Fig 2B). The percentage of cortisol responders did not differ by gender (χ^2^(1, *N* = 39) = 0.140, *p* = .71).

#### Physiological responses

Physiological measurements during MRI scanning included collection of participants’ heart rate using a photoplethysmograph placed on the left index finger, and respiratory data using a respiration belt placed around the chest. The physiological data were transformed to power spectral densities (PSD) using Welch’s method (pwelch). We investigated whether PSD values differed between the three resting-state measurements of the frequencies that coincided with the resolution of MRI scanning (TR = 2s, resulting in a frequency window-of-interest 0.0075–0.25 Hz).

### Data acquisition

Data were acquired on a 3-T Siemens Magnetom Allegra head scanner (Siemens Medical System, Erlangen, Germany). T1-weighted anatomical images were obtained using an MPRAGE sequence with 192 slices and 1 x 1 x 1 mm voxel size covering the whole-brain (repetition time (TR) = 2250 ms, echo time (TE) = 2.6 ms, flip angle = 9°)The anatomical images were obtained from each participant during the iMAST. Functional T2*-weighted resting-state images were acquired using a standard echo-planar imaging (EPI) sequence (TR = 2000 ms, TE = 30 ms, flip angle = 90°, 32 slices, 180 volumes, 3 x 3 x 3 mm). Additionally, a negative slice tilt (30°) was used to minimize inhomogeneity artefacts [32].

### fMRI data pre-processing

The fMRI data were pre-processed and analysed using BrainVoyager QX 2.8 software (Brain Innovation, Maastricht, the Netherlands) [33]. For functional datasets, the first two volumes of each complete time series were discarded because of saturation effects. Pre-processing of the functional data included removing the first two volumes, correction for slice time differences using sinc interpolation, 3D motion correction using sinc interpolation, spatial smoothing using a 4 mm full-width-at-half-maximum isotropic Gaussian Kernel and linear trend removal. Individual functional datasets were co-registered with the 3D anatomical data, normalized in Talairach space (1x1x1mm), and were averaged to create a group-based mask to exclude voxels belonging to the ventricles or tissue outside of the brain for further analysis.

### Selection of amygdala seed from implicit emotion task

**Fig 3:**
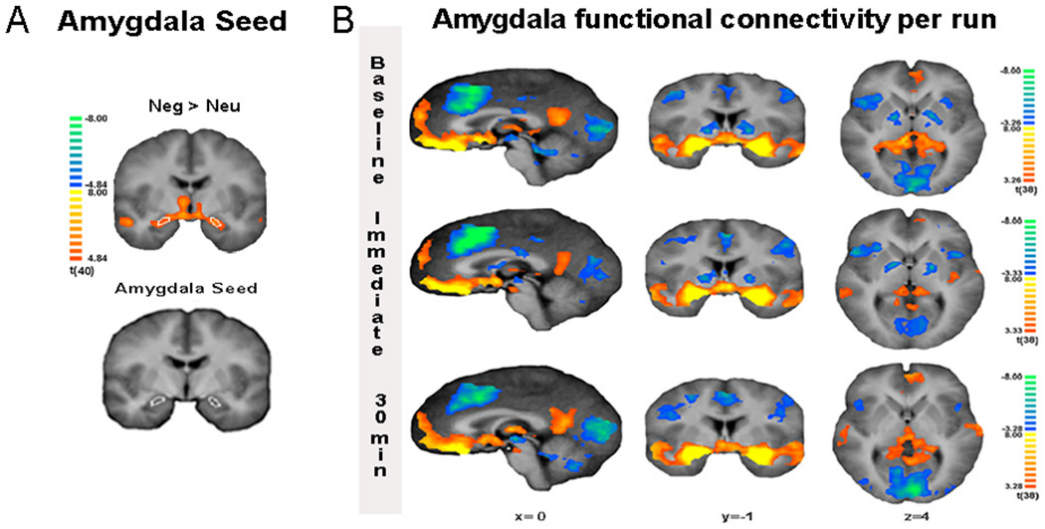
Implicit emotion task and functional connectivity of the amygdala. **A)** The extraction of the amygdala seed. Statistical maps and amygdala ROI selection for the contrast negative > neutral (FDR correction threshold of *q* = .001) overlaid on the anatomical average of the participants. **B)** Amygdala resting state functional connectivity t-maps per run overlaid on the anatomical average of the participants (FDR correction threshold of *q* = .005).

To select amygdala seeds, we investigated the statistical contrast negative > neutral of the implicit emotion task using a whole-brain random effects general linear model (RFX-GLM). The boxcar for the sequences of image presentation was convolved using a two-gamma hemodynamic response function to account for delay of the hemodynamic signal. The resulting contrast map was thresholded using a statistical (*q*(FDR) = 0.01) and cluster-size threshold (i.e. minimum cluster size of 216 mm^3^ estimated by a stochastic procedure of the statistical map that incorporated the estimated spatial smoothness of the target statistical map with 1,000 Monte Carlo simulations) [33]. We selected a homogeneous voxel cluster in the left (center coordinate in Talairach space: x, y, z = -20.82, -5.27, -13.10; 253 mm^3^) and the right amygdala (21.73, -6.28, -11.80; 305 mm^3^) as corresponding amygdala seeds (see Fig 3A).

### Functional connectivity analysis

We used seed-based correlation analysis (SCA) to assess amygdala-based whole-brain functional connectivity of the three resting-state measurements. The analysis was performed using NeuroElf (an MR imaging analysis toolbox, www.neuroelf.net) and custom routines in Matlab (Mathworks, Inc.). Data of the left and right amygdala were collapsed because the correlation between the time courses was high for all three resting state runs (*r*>.61).

We first estimated amygdala-seeded functional connectivity for each participant separately (first-level analysis), which then served as input to a multi-subject statistical analysis (second-level) using an ANOVA model with between-subject factor cortisol responder type and within-subject factor resting state run. Following previous studies and recommendations [34,35], we removed a number of nuisance covariates from the fMRI signal using linear regression before correlating the time series with the amygdala seed. The following parameters were included as regressors: six 3D head motion parameters and their first derivatives, mean signal from the ventricles and white matter, and the global signal. All covariates were Z-normalized to equalize variance. In addition, we added signal oscillations at a frequency above 0.1 Hz (sine-cosine pairs) for low-pass filtering of the time series. Note that there is a debate as to what the global signal represents [36], and whether regressing out the global signal is a valid approach [37] but see [38]. We regressed out the global signal because our focus was on network-specific synchronization of activity unrelated to unspecific global brain activity differences induced by the stressor. After cleaning of the fMRI signal, we then correlated (Pearson’s *r*) the blood oxygenation level dependent (BOLD) time course extracted from the amygdala seed with the cleaned time series from all other brain voxels for each participant.

The first-level voxel-by-voxel correlation coefficients *r* were then transformed to normality using Fisher’s *Z* normalization and entered into a second-level analysis to study rsFC changes for acute stress and early recovery using a whole-brain random effects mixed-model ANOVA. To summarize the data across all participants, we calculated multi-subject maps for each of the resting state measurements using a mass-univariate (i.e., voxel-by-voxel) one-sample *t*-test (thresholded for visualization using a statistical *q*(FDR) = .005; cluster-size threshold 216 mm^3^). The resulting F-maps were thresholded using a statistical threshold (*p*<.025, uncorrected) and corrected for multiple comparisons at the 3D cluster level (cluster-level threshold *p* = 0.05, estimated minimum cluster-size = 216 mm^3^).

Connectivity changes as a function of time intervals around stress induction were post-hoc analyzed using a Bonferroni corrected *t*-test on the differences scores: acute stress (run 2—run 1) and early recovery (run 3—run 2). Differences between cortisol responders and non-responders were analyzed using a mixed model with cortisol responder type as between-subject factor. Additionally, Pearson’s bivariate correlations (Bonferroni corrected, *p*<.01) were computed to investigate to what extent amygdala rsFC changes were linearly associated with subjective and neuroendocrine stress responses.

## Results

### Subjective, neuroendocrine, and physiological stress responses

We used the iMAST as acute stressor to elicit subjective and neuroendocrine stress responses. Subjects rated the iMAST as distressing on a 100mm VAS scale (*M* = 74.2, *SEM =* 3.24; see S1 Table). There were no differences in subjective stress between cortisol responders and non-responders (*F*_(1,37)_ = 0.32, *p* = .57). Neuroendocrine stress responses were defined as salivary cortisol concentration prior to and following the iMAST. For the entire sample, repeated measures ANOVAs revealed significant main effects of Time (6 levels: t_MRI_, t_pre-stress_, t_+0_, t_+10_, t_+30_, t_+40min_: *F*_(2.30,85.04)_ = 4.94, *p* = .007) with a significant increase of cortisol up until t_+40_ (all *ps*<.05; see S1 Table & Fig 2). Comparison of the saliva sample at placement (t_MRI_) with the sample immediately before the iMAST (t_pre-stress_) demonstrated that lying in the scanner did not induce any changes in cortisol (*p*>.99; see also 26).

The repeated measures ANOVAs with Time as within-subject factor for pulse and respiratory power spectral densities (PSDs) were non-significant for all frequencies in the window-of-interest (*p*s>0.05, FWE-corrected) and for the low-frequency range (< 0.1 Hz; pulse: *F*_(2, 72)_ = 1.66, *p* = 0.20; respiratory: *F*_(2, 72)_ = 1.79, *p* = 0.17; see S1 Fig). This indicates that the observed differences between the runs in functional connectivity are not due to differences in physiological responses that are known to influence the BOLD signal in the resting state low-frequency range [39,40].

### Amygdala functional connectivity

We implemented SCA on the three runs of eight minutes of resting state data to investigate how acute stress changes the amygdala connectivity during acute stress and early recovery (see S2 Fig).

First, brain regions that were functionally coupled to the amygdala were identified for each resting state run separately (one-sample *t-*tests, minimum cluster size of 216 mm^3^, see Fig 3B). Regions showing significant functional connectivity with the amygdala in all three runs include the lateral frontal pole, lateral orbitofrontal cortex, medial frontal cortex, anterior dorsal cingulate cortex, dorsal and ventral posterior cingulate cortex, hippocampus, hypothalamus, insula, temporal pole, superior temporal gyrus, midbrain, and visual cortex. The majority of these regions have been previously described in rsFC of the amygdala [10,41,42].

### Amygdala functional connectivity during acute stress

To identify amygdala rsFC changes characterizing acute stress, we subtracted the baseline from the reactivity measurement (run 2—run 1). A whole-brain analysis revealed that seven clusters showed a significant effect of acute stress during acute stress. A reduced amygdala rsFC was found with the left ventrolateral prefrontal cortex (vlPFC), left and right ventral posterior cingulate cortex (vPCC), culmen, and bilateral cuneus while the amygdala rsFC was enhanced with the right parahippocampal gyrus immediate after acute stress (see Table 1 for statistical values).

**Table 1:** Amygdala functional connectivity results based on whole brain analysis of the two contrasts of interest: acute stress (run 2—run 1) and early recovery (run 3—run 2).

Region	Peak Voxel coordinates			Statistical values		Average ConnectivityFisher’s Z			Description of results
*Amygdala -*	*x*	*y*	*z*	*t (p)*		*Run 1*	*Run 2*	*Run 3*	
**Acute stress (run 2–1)**									
Ventrolateral Prefrontal Cortex(vlPFC)	-55	22	24	3.60	(=.001)	-.142	-.028	-.050	↓
Ventral Posterior Cingulate Cortex (vPCC)	23	-35	7	-5.00	(<.001)	.231	.076	.150	↓
	-25	-41	6	-3.98	(<.001)	.203	.052	.127	↓
Parahippocampal Gyrus	23	-29	-22	3.69	(=.001)	.135	.260	.238	↑
Cuneus	-7	-83	9	4.50	(<.001)	-.192	-.093	-.230	↓
Culmen	11	-38	-23	4.19	(<.001)	-.220	-.057	-.134	↓
**Recovery (run 3–2)**									
Dorsolateral Prefrontal Cortex (dlPFC)	26	25	36	3.55	(=.001)	-.187	-.247	-.101	↓
Ventral Anterior Cingulate Cortex (vACC)	-1	25	24	3.40	(=.002)	-.188	-.211	-.086	↓
Dorsal Anterior Cingulate Cortex (dACC)	-1	42	0	2.98	(=.005)	.164	.088	.194	↑
Presupplementary Moter Area (preSMA)	-4	10	48	3.33	(=.002)	-.297	-.342	-.233	↓
Anterior Hippocampal Complex	29	4	-18	4.92	(<.001)	.571	.507	.634	↑
Cuneus	-7	-83	9	-5.00	(<.001)	-.192	-.093	-.230	↑

Note: For each significant pairwise connection a description of the results are presented as either an increase (↑) or a decrease in connectivity strength (↓). The peak voxel coordinates are Talairach coordinates in mm.

**Fig 4:**
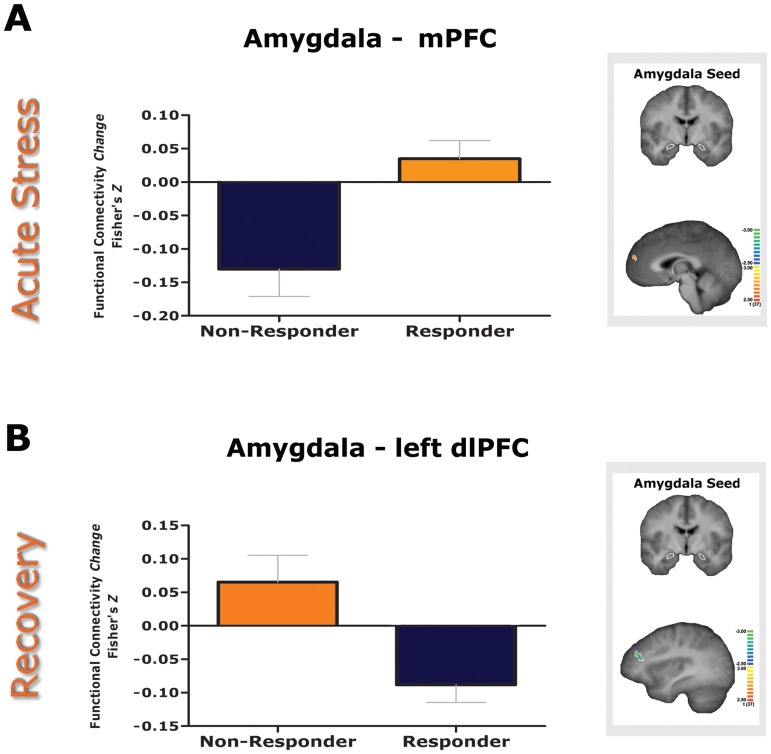
Amygdala rsFC differences between cortisol responders and non-responders during the two stress phases. **A)** Acute stress (run 2–1): The group contrast revealed an opposing effect of stress on the amygdala—mPFC rsFC by cortisol responders (**↑**) and non-responders (**↓**). **B)** Recovery (run 3–2). The group contrast revealed an opposing effect of stress on the amygdala—dlPFC (shown in the figure), culmen and dACC rsFC by cortisol responders (**↓**) and non-responders (**↑**) while the opposite pattern was found for the rsFC with the anterior hippocampal complex and parahippocampal gyrus responders (**↑**) and non-responders (**↓**).

The group contrast of cortisol responders versus non-responders revealed a significant main effect in the mPFC, reflecting an opposing effect of stress on the amygdala rsFC by cortisol responders (enhanced) and non-responders (reduced) (see Fig 4A & Table 2).

**Table 2:** Resting state functional connectivity results of the non-responder versus cortisol responder comparison during the two stress phases.

*Region*	Peak Voxel Coordinates			Statistical Values		Average Connectivity Change Fisher’s *Z* (SEM)	
*Amygdala -*	*x*	*y*	*z*	*t (p)*		*Non-responders*	*Responders*
**Acute stress (run 2–1)**							
Medial Prefrontal Cortex (mPFC)	-1	53	15	-3.35	(=.002)	-.130 (.04)	.035 (.03)
**Recovery (run 3–2)**							
Dorsolateral Prefrontal Cortex (dlPFC)	-34	37	27	3.18	(=.003)	.065 (.04)	-.088 (.03)
Dorsal Anterior Cingulate Cortex (dACC)	-4	7	39	3.36	(=.002)	.142 (.05)	-.046 (.03)
Anterior Hippocampal Complex	33	-29	-9	-4.01	(<.001)	-.085 (.03)	.066 (.02)
Parahippocampal Gyrus	-19	-17	-15	-4.31	(<.001)	-.077 (.04)	.091 (.02)
Culmen	-17	-41	-24	4.23	(<.001)	.105 (.03)	-.076 (.02)

Note: The peak voxel coordinates are Talairach coordinates in mm.

### Amygdala functional connectivity during recovery of stress

To identify amygdala rsFC changes characterizing early recovery of stress, we subtracted the reactivity from the 30min rs-fMRI run (run 3—run 2). The whole-brain analysis revealed an effect on the early recovery of acute stress in six clusters. Early acute stress recovery was characterized by reduced amygdala rsFC with the ventral anterior cingulate cortex (vACC), right dorsolateral prefrontal cortex (dlPFC) and the left presupplementary motor area (preSMA) while the amygdala rsFC was enhanced with the dorsal anterior cingulate cortex (dACC), right anterior hippocampal complex consisting of the amygdala and hippocampus, and bilateral cuneus (see Table 1).

The group contrast of cortisol responders versus non-responders revealed a significant main effect in five areas reflecting the opposing effect of stress recovery on the amygdala rsFC. Opposite to non-responders, cortisol responders were characterized by enhanced amygdala connectivity with the anterior hippocampal complex and parahippocampal gyrus, and reduced connectivity with left dlPFC, dACC, and culmen (see Fig 4B & Table 2 for statistical values).

### Association of amygdala rsFC with neuroendocrine and subjective measures

**Fig 5:**
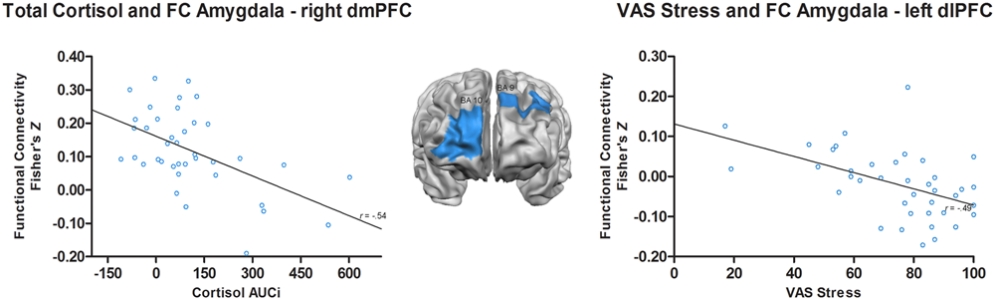
Association of amygdala rsFC with total cortisol and subjective stress. The left scatterplot illustrates the negative correlation between total cortisol and strength of the baseline amygdala—right dmPFC connectivity. The right scatterplot illustrates the negative correlation between subjective stress and strength of the reactivity amygdala—left dlPFC connectivity.

Pearson’s bivariate correlations (*p*<.01) were computed to investigate to what extent amygdala rsFC was linearly associated with total neuroendocrine and subjective stress responses. Baseline (run 1) amygdala—right dmPFC functional connectivity was negatively correlated with the total cortisol concentration AUCi (*r* = -.54, *p*<.001). Additionally, amygdala—left dlPFC functional connectivity immediately after stress (run 2) was negatively correlated with subjective stress (*r* = -.49, *p* = .002; see Fig 5).

## Discussion

The current study investigated how acute stress affects the temporal trajectory of the amygdala’s rsFC. Using the iMAST [26], we obtained strong increases in cortisol levels, enabling us to explore the role of neuroendocrine stress responses in amygdala based rsFC. A particular strength of this study is that it deals with the role of glucocorticoids in the amygdala connectivity changes during two phases that follow stress exposure: acute stress and early recovery.

First, acute stress was characterized by enhanced rsFC between the amygdala and parahippocampal gyrus. The parahippocampal gyrus has a strong functional connection with the amygdala [43] and has been associated with appraisal and perceived stress [44,45]. Furthermore, the negative rsFC with the vlPFC, cuneus and culmen and the positive rsFC with the PCC decreased immediately after acute stress. Our results fit with previous reports of increased perfusion in the cuneus at rest immediately after exogenous cortisol administration [46] as well as enhanced visual processing after acute stress [17,47] and during the perception of fear-relevant images [48] optimizing the detection of threats. In line with this reasoning, we also found decreased PCC connectivity during acute stress. This can be regarded as a sign of deactivation in the default mode network (DMN), which is required for focused attention [49]. Thus, decreased PCC connectivity likely promotes focused attention and optimizes threat detection. Moreover, the decreased amygdala—PCC coupling during acute stress mirrors the findings of Veer and colleagues [10] of an enhanced coupling during recovery, suggesting a dynamic connectivity pattern modulating threat detection and attention when it is crucial.

Interestingly, during acute stress, the functional connectivity between the amygdala and mPFC was in opposite direction in cortisol responders and non-responders, with an increased connectivity in responders. This finding is seemingly at odds with the absence of a rapid effect of exogenous cortisol administration on the functional connectivity of the amygdala and mPFC during an emotional processing task [50]. One explanation for the disparity may be that exogenous cortisol administration activates almost exclusively the HPA-axis while a psychological stressor also increases the release of other hormones like catecholamines [51,52]. Animal studies have shown that the mPFC activates behavioural and neuroendocrine systems to acute stress [53]. Our results support the notion that the HPA response to stress and stress integrative functions are regulated by forebrain circuits [5,54]. Our findings are also in line with previous human studies demonstrating that the mPFC modulates the amygdala response during regulation of autonomic and affective responses [18,21,22].

Second, recovery from the stressor was characterized by a reduced negative connectivity between the amygdala and the ventral ACC and preSMA and enhanced negative rsFC with the cuneus. The vACC is thought to contribute to adaptive emotion regulation, and specifically, to down-regulate limbic regions involved in generating emotional responses [55,56]. In line with this, reduced connectivity between the amygdala and vACC has been found in stress-related psychiatric disorders [57,58]. Additionally, we found that early recovery from the stressor was characterized by changes in connectivity of the amygdala with the dACC and left dlPFC. These changes in amygdala connectivity interacted with the cortisol response, as evidenced by our cortisol responder versus non-responder analysis. Cortisol responders displayed reduced amygdala—dlPFC and amygdala—dACC functional connectivity. The dACC is part of an intrinsic salience network that regulates adaptive behavior in response to environmental stimuli that produce autonomic reactions [59–61]. Our results extend previous reports of enhanced amygdala rsFC with the dACC during acute stress [9,11] by directly comparing cortisol responders and non-responders, and by directly comparing the rsFC immediately and 30min after the stressor. Together, these findings suggest a dynamic connectivity pattern regulating the autonomic response that is dependent on the stress phase. Participants with higher HPA-axis reactivity displayed a reduced amygdala-left dlPFC functional connectivity. Functional asymmetries in the PFC are said to be relevant to stress adaptation, with the left PFC being involved in effective coping and preventing small stressors from becoming significant ones [53,62,63]. Thus, the decreased amygdala—left dlPFC rsFC in cortisol responders might suggest less effective coping and possibly increased vulnerability to stress.

Enhanced emotional memory consolidation is a well-known long-term adaptive response after a stressor, and the connectivity between the amygdala and hippocampus is thought to regulate this effect [64,65]. In line with this, we found enhanced connectivity between the amygdala and anterior hippocampal complex during stress recovery. Interestingly, we found that this change in rsFC connectivity was dependent on glucocorticoid levels, as demonstrated by an opposite direction of the functional connectivity between the amygdala and anterior hippocampal complex in cortisol responders and non-responders. In cortisol responders, recovery was characterized by enhanced amygdala rsFC with the left parahippocampal gyrus and right hippocampal complex. This is in accordance with the view that returning glucocorticoids reactivate the hippocampus after initial stress exposure [24].

Finally, an investigation of the relation between baseline amygdala rsFC and neuroendocrine measure of stress revealed that enhanced amygdala—right dmPFC connectivity was associated with a lower total increase of cortisol in response to the iMAST. This result is consistent with the findings from rodent studies indicating the importance of the dmPFC in reducing HPA activation [53]. Interestingly, subjective stress was inversely associated with amygdala—left dlPFC reactivity rsFC. Immediately after acute stress, stronger amygdala—left dlPFC connectivity was associated with less experienced stress. These findings are in agreement with the idea that the connectivity between the left PFC and amygdala plays a central role in the down-regulation of negative affect [66] and with the proposed role of asymmetrical activation of the prefrontal cortex in stress adaptation via biased motivational processing.

Several issues deserve consideration. First, in light of the debate regarding a possible influence of tasks on the rsFC in a subsequent resting state measurement [67, 68] but see [69, 70], we investigated the influence of an emotional working memory task on rsFC changes during recovery. Per participant, the time course of the amygdala was extracted in the emotional working memory contrast negative > neutral using a whole-brain random effects general linear model. The individual beta values from the amygdala in the emotional working memory task were then correlated with the individual amygdala rsFC Fisher’s Z transformed correlation coefficients *r* of areas where a significant effect between run 2 and 3 was found (i.e., dlPFC, ventral and dorsal ACC, presupplementary motor area, anterior hippocampal complex, parahippocampal gyrus, cuneus, and culmen). All correlations were non-significant. Moreover, by using the cortisol responder versus non-responder contrast, the influence of the emotional working memory task was curtailed, because all participants had to perform the task. Second, the current study did not include a no-stress control condition. To further investigate the influence of task-engagement, and for future research aiming to investigate stress mechanisms in the brain, a no-stress placebo version of the iMAST is needed. The no-stress placebo version would be similar to the iMAST in terms of physical and mental load as well as its duration, but without eliciting stress reactions [71]. Third, the current study investigated the early recovery phase after stress induction controlled by non-genomic glucocorticoid actions. It would be interesting to also investigate the delayed genomic effect of acute stress on changes in amygdala connectivity by including resting state measurements during a longer period after the stress induction. Glucocorticoids play an important role in both the onset and the termination of the stress–response via the MR and GR. The membrane-bound MR mediates the onset of the stress response while the GR terminates the stress response and facilitates behavioural adaptation [7]. Future studies may extend the current study’s test of time-dependent effects of glucocorticoid actions by focusing on the different temporal profiles of the MR and GR (e.g., by pharmacologically blocking the GR or by studying MR polymorphisms that affect the functioning of the MR receptor) and by investigating how these two receptor types mediate the effect of cortisol stress responses on the resting state functional connectivity during the different phases after a stressful experience. Finally, although the amygdala rsFC provides a valuable framework to study the effects of stress, investigating in a more exploratory fashion the temporal dynamics of the entire cortex could yield a comprehensive description of brain functional architecture post-stress.

In sum, this study demonstrates that acute stress and early recovery thereof are associated with changes in the functional connectivity of the amygdala network, which is most probably regulated by stress-induced neuroendocrine levels. The early phase after acute stress is characterized by changes in connectivity strength of the amygdala with areas involved in emotional significance, threat detection and HPA-axis regulation. The period after removal of the stressor was characterized by connectivity changes in areas involved in emotion regulation, coping, and suppression of negative affect. Dysfunction of the HPA-axis regulation and impaired coping abilities have been implicated in a variety of psychiatric disorders (e.g., depression, anxiety). Defining stress-induced neuronal network changes is highly relevant for developing treatments that target abnormal neuronal activity. Our results suggest that the left dlPFC might be a target area of brain activity based treatments to promote recovery and stress adaptation. Moreover, repeated resting-state assessments may prove valuable for further investigations of intrinsic post-stress brain activation changes so as to further delineate the temporal trajectory during which cortisol affects specific brain connectivity patterns.

## Supporting Information

S1 Fig Power spectral densities (PSDs) of the physiological responses. Upper panel: Mean PSDs of the pulse rate (black line) and respiration (grey line) across all participants and conditions. For visualization the frequency range is truncated to 1 Hz. Grey area represents the frequency window coinciding with the scanner resolution of TR = 2 s (i.e., 0–0.25 Hz). Lower panels: Mean (± *SEM* area) PSDs of pulse rate (left) and respiration (right) for the scanner-relevant frequency range of the three resting-state measurements. ANOVAs across the plotted frequencies were not significant (all corrected *p*s>0.05).
(TIF)

S2 Fig Overlap between amygdala resting state functional connectivity maps. The overlap with the baseline measurement (i.e., run 1) is shown in purple in the pairwise maps. The amygdala seed used for the analysis is drawn in white. Statistical maps (FDR correction threshold of *q* = .005) are overlaid on the anatomical average of the participants. In the coronal view, the left side of the brain corresponds to the right hemisphere and vice versa.
(TIF)

S1 File Supporting Information. (DOC)

S1 Table Means (± SEM) of subjective stress and cortisol (untransformed values). Grey areas indicate values used for calculation of the area under the curve (AUCi).
(DOCX)
