# Supplementary figures and images for: Temporal Dynamics of Stress-Induced Alternations of Intrinsic Amygdala Connectivity and Neuroendocrine Levels
Source: PLoS One. 2015 May 6;10(5):e0124141. doi: 10.1371/journal.pone.0124141 (PMC4422669; doi:10.1371/journal.pone.0124141)

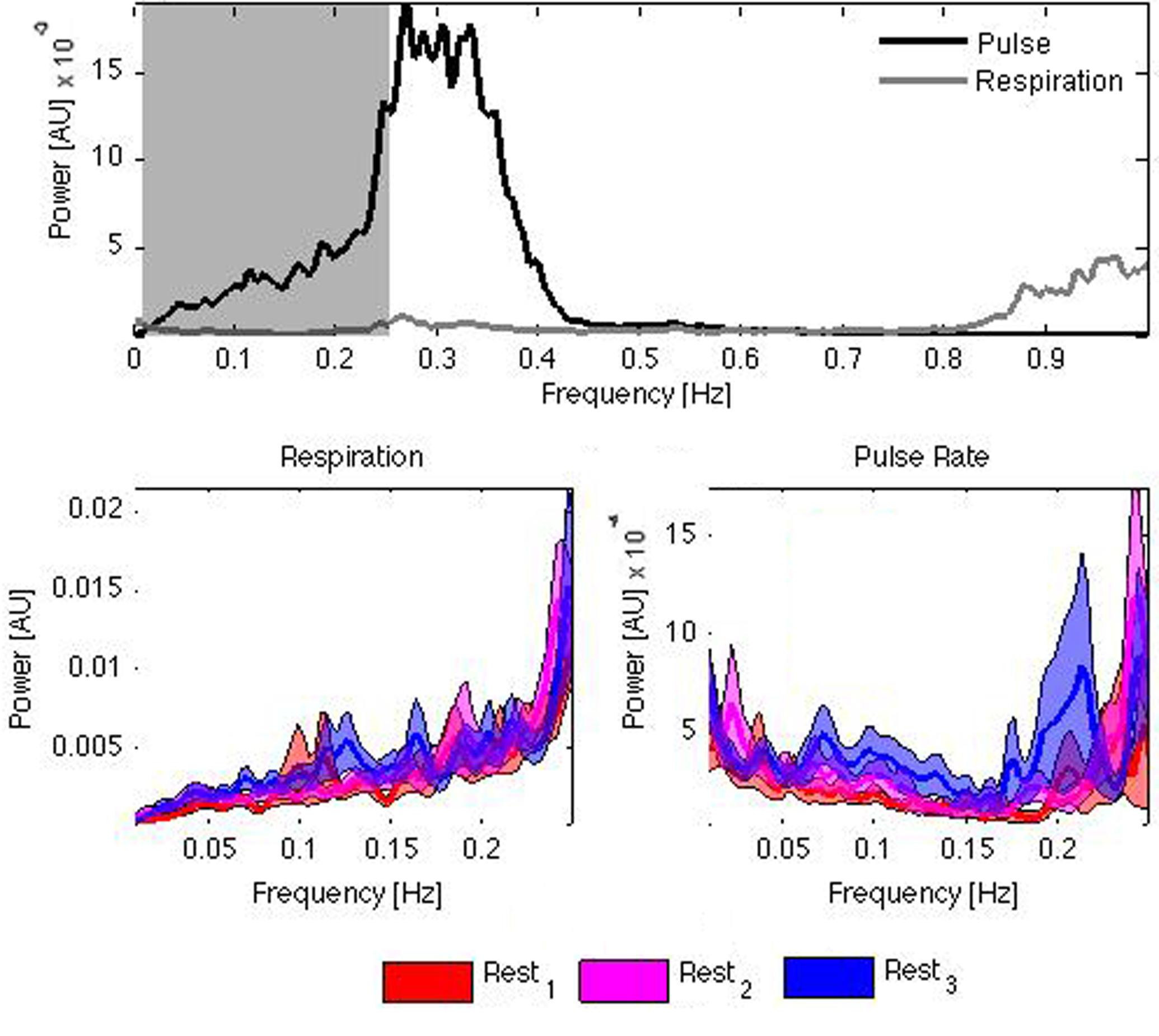

Supplement: S1 Fig — Upper panel: Mean PSDs of the pulse rate (black line) and respiration (grey line) across all participants and conditions. For visualization the frequency range is truncated to 1 Hz. Grey area represents the frequency window coinciding with the scanner resolution of TR = 2 s (i.e., 0–0.25 Hz). Lower panels: Mean (± SEM area) PSDs of pulse rate (left) and respiration (right) for the scanner-relevant frequency range of the three resting-state measurements. ANOVAs across the plotted frequencies were not significant (all corrected ps>0.05). (TIF) [file pone.0124141.s001.tif]

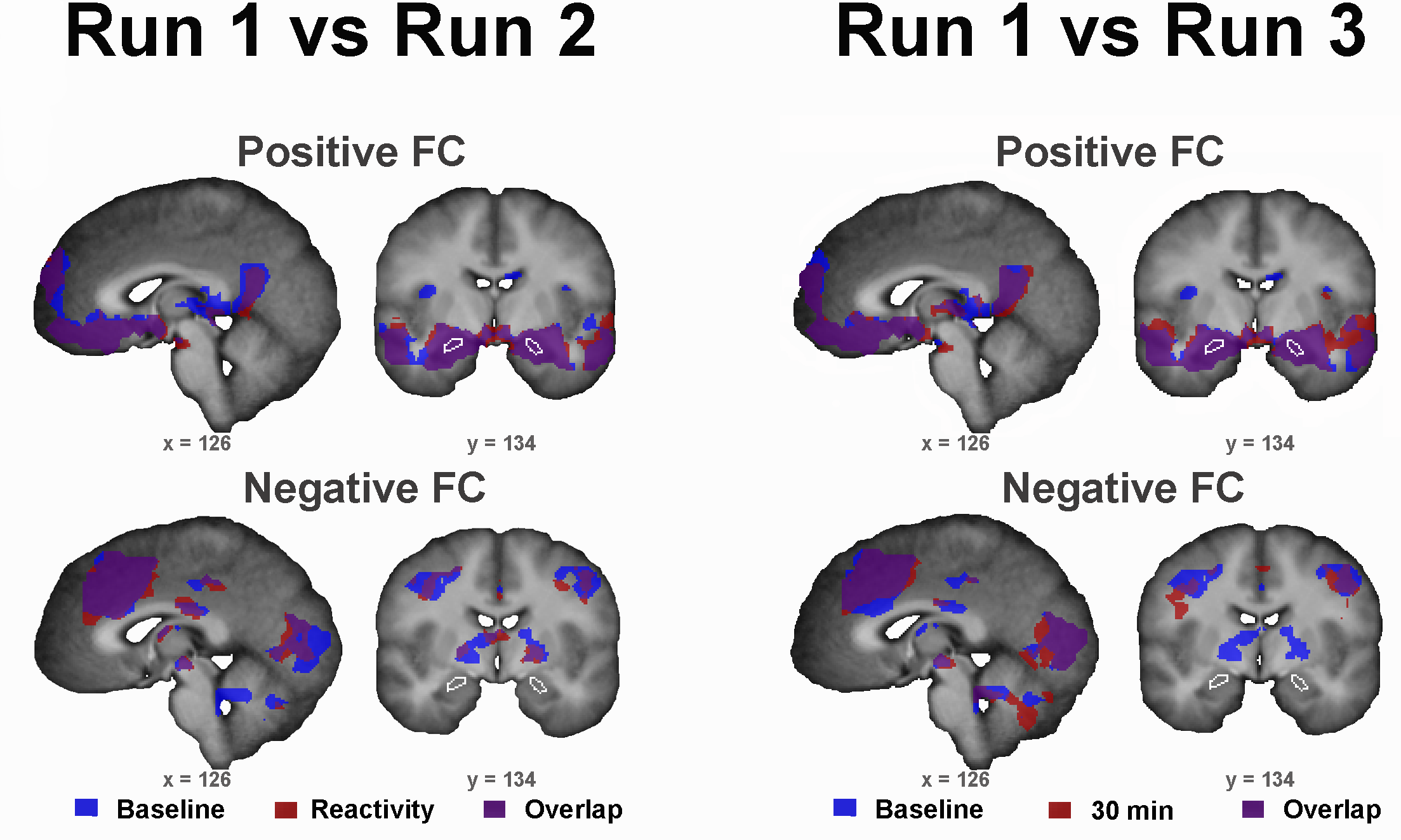

Supplement: S2 Fig — The overlap with the baseline measurement (i.e., run 1) is shown in purple in the pairwise maps. The amygdala seed used for the analysis is drawn in white. Statistical maps (FDR correction threshold of q = .005) are overlaid on the anatomical average of the participants. In the coronal view, the left side of the brain corresponds to the right hemisphere and vice versa. (TIF) [file pone.0124141.s002.tif]
