# Supplementary material for: Temporal Dynamics of Stress-Induced Alternations of Intrinsic Amygdala Connectivity and Neuroendocrine Levels
Source: PLoS One. 2015 May 6;10(5):e0124141. doi: 10.1371/journal.pone.0124141 (PMC4422669; doi:10.1371/journal.pone.0124141)
Supplement: S1 File — (DOC) [file pone.0124141.s003.doc]

**S1 File: Supplemental Information**

**Supplemental Experimental Procedures**

**Participants**

Participants were excluded if they had a history of psychiatric, neurologic, cardiovascular or neuroendocrine disease, were considered heavy smokers (i.e., more than 15 cigarettes/day), used medication that affects the ANS or HPA axis, were regular drug users, or had a body mass index (BMI; weight in kg / (height in m)^2^) outside the 18-30 range. An additional exclusion criterion was prior experience with the non-imaging Maastricht Acute Stress test [MAST; 1]. Moreover, for women the use of oral contraceptives served as an inclusion criterion to reduce variability in cortisol responses related to hormonal alterations throughout the menstrual cycle phase [e.g., 2]. Pregnancy or lactation, on the other hand, served as additional exclusion criteria for women.

**Stress Induction Equipment and Procedures**

Cold pressor stress was generated with an fMRI compatible 30x30mm Medoc Pathway advanced thermal stimulator (ATS) thermode (Medoc Ltd, Ramat Yishai, Israel) placed on the left volar forearm. In total, 5 cold pressor stress stimuli of 2°C were applied with a variable duration of 30, 60 or 90 s. In between the cold pressor trials, participants had to engage in mental arithmetic challenges, i.e., counting backwards as fast and accurately as possible in steps of 17 starting at 2043 for 45, 60 or 90 s. Each time they counted too slowly or made a mistake, they received negative feedback via the intercom (i.e., to count faster or start over at 2043). During these mental arithmetic trials, the ATS was set at 25°C. However, to increase the unpredictability of the task, during each of the mental arithmetic trials, one, two or three hot pulses (49°C) of 10 s occurred without warning.

**Neuroendocrine Stress Responses**

The timing of saliva sampling was based on previous work showing that cortisol typically peaks 20 to 40 min after stress onset [e.g., 3]. Practically, for each sample collection, participants were returned to the home position of the scanner, and a research assistant placed the Salivette in the mouth of the participant using sterile plastic tweezers. To facilitate sample collection and to minimize movement, this procedure was trained extensively beforehand during a simulation scan in a dummy MRI scanner.

Saliva samples were stored at -20°C immediately upon collection. Cortisol levels were determined from the saliva samples using a commercially available kinetic reaction assay (Salimetrics, Penn State, PA) and luminescence immune assay kit (IBL, Hamburg, Germany), respectively. Mean intra- and inter-assay coefficients of variation are typically less than 5% for the cortisol analyses.

**Procedure**

All testing took place between 12:30 and 18:00h to control for the circadian rhythm of cortisol. Participants were instructed to refrain from eating, exercising extensively, or drinking anything but non-sparkling water two hours prior to the test session. Upon arrival, written informed consent was obtained from the participant. Next, a saliva sample was taken with the explicit but bogus instruction that it would be immediately assayed to check whether they had adhered to our instructions not to eat, drink, etc. In actual fact, the sample was disposed of without being analysed. This procedure was followed to promote truth-telling behaviour when participants were subsequently asked whether they had adhered to all pre-experimental instructions. If participants indicated to have violated these instructions, the session was terminated and rescheduled. For each measurement, participants were instructed to place the Salivette on the same side of their mouth and not to chew on it [4]. Next, participants received a standardized lunch (a sandwich and 0.5l non-sparkling water) and were asked to complete several questionnaires. Thereafter, and for purposes unrelated to the aims of the current report, electroencephalography (EEG) measurement was prepared and resting EEG activity was measured during 8 min (data to be reported elsewhere). After completion of the scanner session, participants provided ratings of the stimuli used in the emotional reactivity task.

**Implicit emotion task**

The 84 pictures were presented for 1800 ms and the indoor/outdoor decision was made within 1800 ms after picture presentation. Pictures were interspersed with a variable inter-stimulus interval (ISI) (2, 4, 6 or 8 s) during which a fixation cross was presented. The presentation order of the pictures was semi-randomized so that no more than three pictures of one condition appeared consecutively in order to avoid mood state induction [5].

**Supporting References**

1. Smeets T, Cornelisse S, Quaedflieg CWEM, Meyer T, Jelicic M, Merckelbach H (2012) Introducing the Maastricht Acute Stress Test (MAST): a quick and non-invasive approach to elicit robust autonomic and glucocorticoid stress responses. Psychoneuroendocrino. 37: 1998-2008.

2. Kudielka BM, Hellhammer DH, Wust S (2009) Why do we respond so differently? Reviewing determinants of human salivary cortisol responses to challenge. Psychoneuroendocrino. 34: 2-18.

3. Dickerson SS, Kemeny ME (2004) Acute stressors and cortisol responses: a theoretical integration and synthesis of laboratory research. Psychol Bull. 130: 355-391.

4. Beltzer EK, Fortunato, CK, Guaderrama MM, Peckins MK, Garramone BM, Granger DA (2010). Salivary flow and alpha-amylase: collection technique, duration, and oral fluid type. Physiol Behav, 101: 289-296.

5. Ritchey M, Dolcos F, Cabeza R (2008) Role of amygdala connectivity in the persistence of emotional memories over time: an event-related FMRI investigation. Cereb Cortex. 18: 2494-2504.

**Legends**

**S1 Fig**. Power spectral densities (PSDs) of the physiological responses. Upper panel: Mean PSDs of the pulse rate (black line) and respiration (grey line) across all participants and conditions. For visualization the frequency range is truncated to 1 Hz. Grey area represents the frequency window coinciding with the scanner resolution of TR = 2s (i.e., 0 - 0.25 Hz). Lower panels: Mean (+- SEM area) PSDs of pulse rate (left) and respiration (right) for the scanner-relevant frequency range of the three resting-state measurements. ANOVAs across the plotted frequencies were not significant (all corrected *p*s > 0.05).

**S2 Fig.** Overlap between amygdala resting state functional connectivity maps**.** The overlap with the baseline measurement (i.e., run 1) is shown in purple in the pairwise maps. The amygdala seed used for the analysis is drawn in white. Statistical maps (FDR correction threshold of *q* = .005) are overlaid on the anatomical average of the participants. In the coronal view, the left side of the brain corresponds to the right hemisphere and vice versa.

**S1 Table** Means (± SEM) of subjective stress and cortisol (untransformed values). Grey areas indicate values used for calculation of the area under the curve (AUCi).
