# Supplementary material for: Temporal Dynamics of Stress-Induced Alternations of Intrinsic Amygdala Connectivity and Neuroendocrine Levels
Source: PLoS One. 2015 May 6;10(5):e0124141. doi: 10.1371/journal.pone.0124141 (PMC4422669; doi:10.1371/journal.pone.0124141)
Supplement: S1 Table — Grey areas indicate values used for calculation of the area under the curve (AUCi). (DOCX) [file pone.0124141.s004.docx]

**Supplemental Data**

**S1 Table** Means (± SEM) of subjective stress and cortisol (untransformed values). Grey areas indicate values used for calculation of the area under the curve (AUCi).

| **Subjective stress** (0-100) | 74.2 | (3.24) |  |
| --- | --- | --- | --- |
| **Cortisol** (nmol/l) |  |  | **CORT-AUCi** |
| t_MRI_ | 9.80 | (1.61) |  |
| t_pre-stress_ | 8.10 | (0.91) | 108.49  (25.63) |
| t_+0_ | 9.40 | (1.12) |  |
| t_+10_ | 11.70 | (1.53) |  |
| t_+30_ | 10.40 | (1.25) |  |
| t_+40_ | 8.80 | (0.84) |  |

*_Note:_* _The CORT-AUCi of responders vs non-responder was significant different (_*_p_* _< .006) while there were no difference s in subjective stress (_*_p_* _= .57) and no gender differences between responder and non-responders (_*_p_*_= .71)._
